# Short-Term Reproducibility of the Inflammatory Phenotype in Different Subgroups of Adult Asthma Cohort

**DOI:** 10.1155/2015/419039

**Published:** 2015-03-04

**Authors:** Sebastian Majewski, Maciej Ciebiada, Mateusz Domagala, Zofia Kurmanowska, Pawel Gorski

**Affiliations:** ^1^Department of Pneumology and Allergy, Medical University of Lodz, Kopcinskiego Street 22, 90-153 Lodz, Poland; ^2^Department of General and Oncological Pneumology, Medical University of Lodz, Kopcinskiego Street 22, 90-153 Lodz, Poland

## Abstract

Inflammatory phenotype classification using induced sputum appears attractive as it can be applied to inflammation-based management of the patients with asthma. The aim of the study was to determine the reproducibility of inflammatory phenotype over time in patients with asthma. In 66 adults asthma was categorized as steroid-naïve (SN, *n* = 17), mild to moderate (MMA, *n* = 33), and refractory treated with oral corticosteroids (RA, *n* = 16). Clinical assessment, skin prick testing, spirometry, and two sputum inductions in 4–6-week interval were done. Inflammatory phenotypes were classified as eosinophilic (EA), consisting of eosinophilic and mixed granulocytic phenotypes, and noneosinophilic (NEA) consisting of paucigranulocytic and neutrophilic phenotypes. During study asthma treatment remained constant. In SN group 25% of patients changed phenotype from EA to NEA and 44% changed phenotype from NEA to EA. In MMA group 26% of patients changed phenotype from EA to NEA and 50% changed phenotype from NEA to EA. In 29% of RA patients inflammatory phenotype changed from EA to NEA and in 22% it changed from NEA to EA. Inflammatory classification, using induced sputum, is not fully reproducible in adults with asthma in short-term evaluation. EA seems to be more stable phenotype across all subgroups whereas NEA remained stable only in RA group.

## 1. Introduction

It is widely accepted that asthma is a heterogeneous disease of the airways, in which many different cells and cellular mediators play a role. Guidelines defined asthma as a chronic inflammatory disorder of the lungs characterized by variable airway obstruction and typical clinical symptoms as cough, wheeze, and dyspnoea [1]. However, assessment of lung function and symptoms does not allow insight into the underlying inflammation of the airways. Development of noninvasive tools to study airway inflammation, such as induced sputum, has facilitated this process, resulting in recognition of apparently distinct patterns of inflammatory phenotypes [2]. The advantage of distinguishing between inflammatory phenotypes in asthma is to identify subgroups of patients who are more likely to respond to individually tailored treatment. Studies have proved that eosinophilic airway inflammation predicts good response to inhaled corticosteroids (ICS), whereas noneosinophilic asthma is less responsive to ICS [3, 4]. Previous studies from different laboratories have reported good reproducibility of induced sputum cell counts [5, 6]. But there are limited and conflicting data on the stability of the phenotype classification in asthma patients. Short-term and long-term stability of sputum inflammatory phenotypes have been reported in two earlier studies [7, 8]. More recent studies show substantial variability in sputum inflammatory phenotypes in both the adults [9–11] and children [12]. Change in asthma control and ICS treatment, as well as environmental exposure to asthma triggers, may affect sputum cellularity and should be considered when evaluating phenotype stability over time.

Inflammation-based asthma management promises improved disease control, providing that inflammatory phenotypes are reproducible and reliable. Under such a condition asthmatics' sputum inflammatory profiles could help guide clinical decisions in personalized medicine approach.

To increase knowledge on stability and possible usefulness of sputum profiles in asthma management we have examined reproducibility of the sputum inflammatory phenotypes over time in the different groups of clinically stable, symptomatic adult asthma cohort.

## 2. Methods

### 2.1. Study Population

Currently nonsmoking adults (lifetime history of smoking < 10 pack-years) with symptomatic asthma, defined according to GINA guidelines [1], with positive reversibility test and/or demonstrated airway hyperresponsiveness in methacholine challenge were studied. Subjects were recruited from the asthmatics referred to the Outpatient Clinic of the Department of Pneumology and Allergy at the Norbert Barlicki Memorial Teaching Hospital No. 1 in Lodz, Poland. They were divided into 3 groups: newly diagnosed, mild steroid-naïve asthmatics (SN group; *n* = 17); mild to moderate asthmatics receiving established inhaled steroid (ICS) treatment (MMA group; *n* = 33); and a group of patients with refractory asthma, diagnosed according to GINA guidelines [1], requiring oral corticosteroid (OCS) treatment to control disease symptoms (RA group; *n* = 16). All recruited patients were in stable condition, defined as a disease without exacerbation for at least 1 month before study enrolment. During the study, maintenance antiasthmatic therapies remained stable and were used by participants as prescribed by their physician. Steroid-naïve asthmatics were using only salbutamol as a rescue medication.

### 2.2. Study Design

This study was approved by the Ethics Committee of the Medical University of Lodz (RNN/61/08/KE). All patients gave informed written consent before start of any study procedure.

All participants underwent clinical assessment, skin prick testing, spirometry and two sputum induction procedures in 4 to 6 weeks' interval on two separate clinic visits. Before sputum induction all subjects were asked to fill in a self-administered Asthma Control Questionnaire (ACQ). All subjects had to be clinically stable between study visits. Asthma exacerbation, defined as worsening of asthma symptoms requiring change in the maintenance antiasthmatic treatment, active smoking, chronic sinusitis, gastroesophageal reflux disease, tuberculosis, neoplasmatic disease were exclusion criteria.

### 2.3. Skin Prick Tests

Skin prick testing (SPT) was performed on the volar surface of the arm on normal skin using commercial extracts (Allergopharma J. Ganzer KG, Reinbek, Germany) according to international guidelines [13]. Subjects were tested to 11 aeroallergens, including* Dermatophagoides pteronyssinus*,* Dermatophagoides farinae*, grass, birch, hazel, alder,* Artemisia*, cat and dog dander,* Alternaria alternate*, and* Cladosporium*, with negative (physiological saline) and positive (histamine) controls. The results were measured 15 minutes after allergen application. The test was considered positive if the mean wheal diameter was ≥ 3 mm than the saline control. Data were excluded if the mean wheal for saline control was ≥ 3 mm, the histamine control was < 3 mm, or if the difference of histamine minus saline was < 3 mm.

### 2.4. Spirometry

Spirometry assessment was performed using Lungtest 1000 spirometer (MES, Cracow, Poland) according to ATS/ERS guidelines [14]. FEV1 (forced expiratory volume in 1 second), FVC (forced vital capacity), and FEV1/FVC % were evaluated. Parameters were presented as % of predicted value.

### 2.5. Asthma Control Questionnaire (ACQ)

ACQ measures the adequacy of asthma control [15] and consists of 7 questions. Five questions refer to most important symptoms (nocturnal symptoms, morning symptoms, limitations of daily activity, dyspnea, and wheezing), one question refers to the use of rescue medication, and one question is about actual FEV1 % of predicted value. The final score of ACQ is the average score of the answers given by the patient and may change from 0 (totally controlled) to 6 (totally uncontrolled). The cut-off point defining well-controlled asthma in this questionnaire for clinical practice is established as 0.75 point. The minimal important difference (MID) that is considered as clinically important for ACQ is 0.5 on the 7-point scale.

### 2.6. Sputum Induction

Sputum induction procedure was performed by a trained technician using the method described previously [16]. Briefly, after salbutamol pretreatment (400 *μ*g), aerosols of hypertonic saline at 3%, 4%, and 5% were each inhaled for 7 min via ultrasonic nebulizer (DeVilbiss UltraNeb 3000, Sunrise Medical Ltd, USA) with an output of 1 mL/min. Patients were asked to cough into container after each cycle. The procedure was monitored by spirometry assessments at baseline and after each saline inhalation. If there was a fall in FEV1 of  ≥ 20% versus baseline, the procedure was discontinued. Fall in FEV1 of 10–19% was an indication to continue the induction with the same concentration of saline.

### 2.7. Sputum Analysis

The sputum was selected from the expectorate and processed within 2 hours as described previously [5, 16]. Selected sputum plugs were dispersed using dithiothreitol (DTT), then the suspension was filtered, and a total cell count of leukocytes and viability was assessed. Cytospins were stained with May-Grünwald-Giemsa. Differential cell counts were performed on 400 nonsquamous cells. Sputum samples with eosinophils >1.9% were defined as eosinophilic. Neutrophilic inflammation was defined as a neutrophil count of >61% [17]. Based on these two cut points phenotypes were classified as eosinophilic, neutrophilic, paucigranulocytic (<1.9% eosinophils and <61% neutrophils), and mixed granulocytic (>1.9% eosinophils and >61% neutrophils). Eosinophilic asthma (EA) consisted of eosinophilic and mixed granulocytic phenotype and noneosinophilic asthma (NEA) consisted of paucigranulocytic and neutrophilic phenotype.

### 2.8. Data Analysis

Results were expressed as mean ± standard deviation (SD). Normally distributed data were analyzed using an unpaired two-sided *t*-test, whereas data without a normal distribution were analyzed by Mann–Whitney *U* test and *χ* ^2^ test. *P* values <0.05 were considered significant. The package Statistica 9.0 (Tulusa, USA) was used for all analyses.

## 3. Results

### 3.1. Study Participants

66 study participants underwent a successful sputum induction on two consecutive clinic visits within 4 to 6 weeks' interval. We enrolled in the study 17 patients with newly diagnosed, mild steroid-naïve asthma (SN), 33 patients with mild to moderate asthma receiving inhaled steroids (MMA), and 16 asthmatics with refractory asthma (RA), requiring oral corticosteroids. The patients' baseline characteristic is presented in Table 1.

Compared to SN and MMA cohorts, RA group was characterized by older age, longer disease duration, and more impaired lung function. During the study disease control assessed with ACQ remained stable (*P* > 0.05 for all groups in comparison with study visits 1 and 2); however the ACQ score was significantly higher in RA group when compared to SN and MMA groups (Tables 2 and 3).

### 3.2. Sputum Cell Analysis

**Figure 1:**
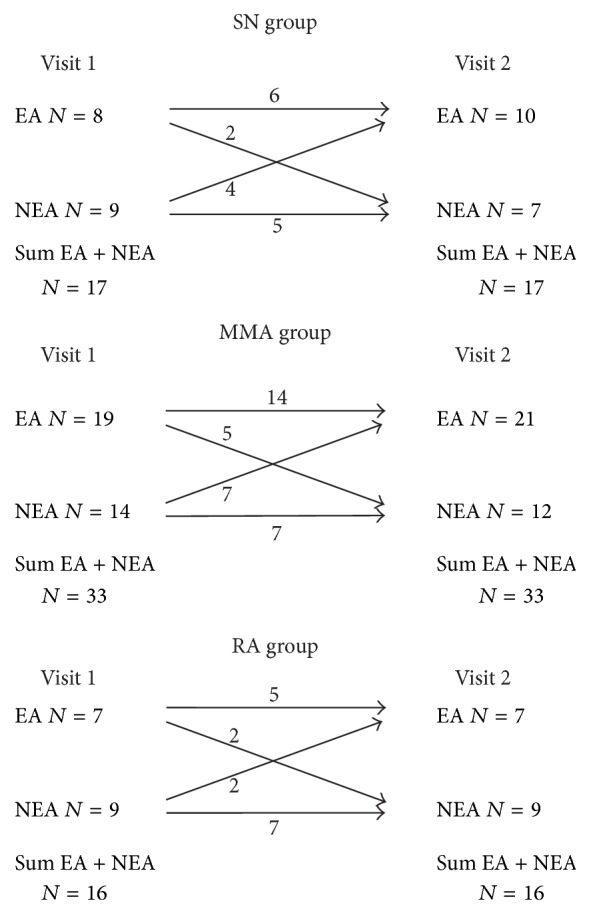
Changes in sputum inflammatory phenotype in studied asthma cohorts: steroid-naïve asthma (SN), mild to moderate asthma (MMA), and refractory asthma (RA).

**Figure 2:**
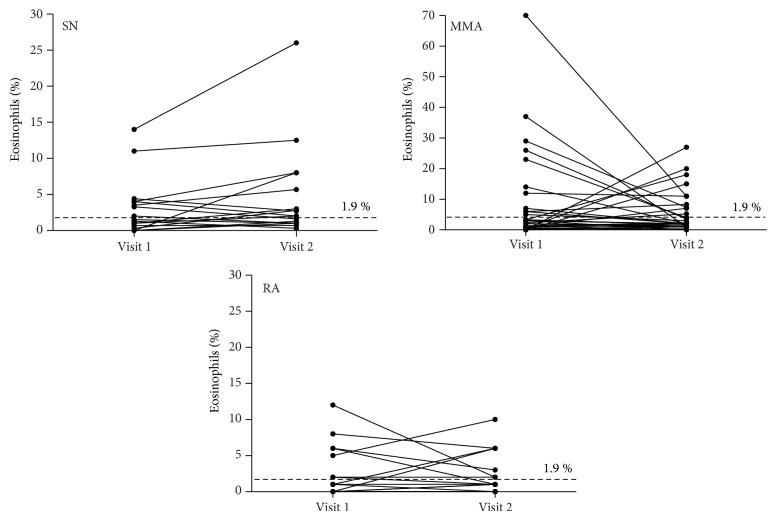
Changes in sputum eosinophils count in studied asthma cohorts: steroid-naïve asthma (SN), mild to moderate asthma (MMA), and refractory asthma (RA).

Based on sputum cell analysis on each of the study visits patients were categorized into one of four inflammatory phenotypes (Tables 2 and 3). At visit 1 EA was diagnosed in 47% of SN patients (8 of 17), 58% of MMA patients (19 of 33), and 44% of RA patients (7 of 16) whereas on visit 2 EA was diagnosed in 59%, 64%, and 44% of patients, respectively (Tables 2 and 3, Figures 1 and 2).

**Figure 3:**
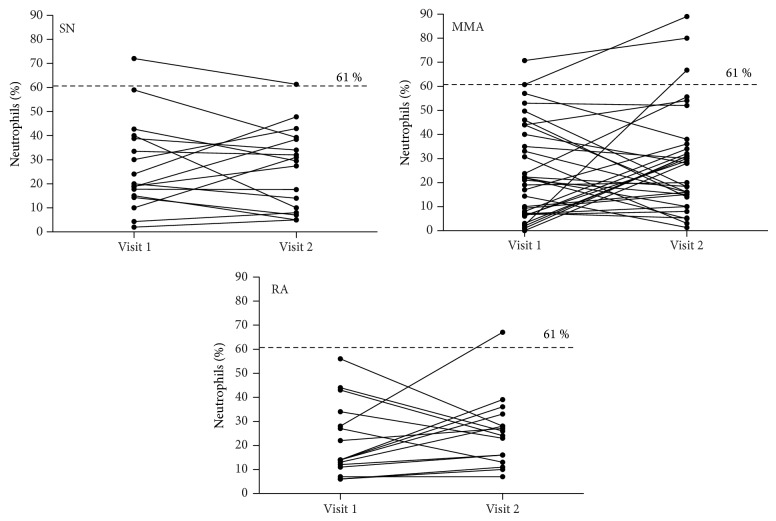
Changes in sputum neutrophils count in studied asthma cohorts: steroid-naïve asthma (SN), mild to moderate asthma (MMA), and refractory asthma (RA).

In SN group 2 of 8 (25%) subjects changed inflammatory phenotype from EA at visit 1 to NEA at visit 2 and 4 of 9 (44%) subjects changed inflammatory phenotype from NEA at visit 1 to EA at visit 2. In MMA group 5 of 19 (26%) subjects changed inflammatory phenotype from EA at visit 1 to NEA at visit 2 and 7 of 14 (50%) subjects changed inflammatory phenotype from NEA at visit 1 to EA at visit 2. In RA group 2 of 7 (29%) subjects classified as EA at visit 1 changed classification to NEA at visit 2 and 2 of 9 (22%) subjects classified as NEA at visit 1 changed classification to EA at visit 2 (Figure 1). Individual changes in sputum eosinophils and neutrophils count are presented in Figures 2 and 3.

## 4. Discussion

Our study demonstrates considerable instability of sputum inflammatory phenotypes over short-term evaluation in different groups of adult asthma cohort. We have observed that EA phenotype seems to be more stable across all groups of asthmatics (only one-fourth of subjects in each group changing phenotype during the study) whereas NEA phenotype remained quite stable only in RA group. Distinction between EA and NEA was not consistent in substantial percentage of studied asthmatics in a short-term evaluation despite no change in asthma treatment and disease control.

Asthma is characterized by intermittent clinical symptoms, variable airway obstruction, and different response to treatment; therefore it is expected that airway inflammation varies between subjects with asthma. Sputum examination in patients with asthma is a noninvasive tool to study airway inflammation [2]. Expectorated or induced sputum in about 50% of asthmatics is rich in eosinophils, but different inflammatory cell profiles have been reported [7, 17], prompting a classification with 4 phenotypes: eosinophilic, neutrophilic, mixed, and paucigranulocytic. Inflammatory phenotyping in asthma may be useful because it relates to treatment response. Studies have shown that eosinophilic airway inflammation predicts better response to ICS, whereas NEA is less responsive to ICS [3, 4]. Such findings warrant clinical implementation of an induced sputum phenotyping as a very attractive tool to help guide asthma treatment. The identification of sputum phenotypes, in most studies of asthma, is generally based on a single sputum sample collected in a cross-sectional setting. This is a potentially significant limitation given that asthma, by definition, is a variable disease. Therefore the stability of the asthma phenotypes, classified according to induced sputum cell profile, is critical to the utility of sputum assessment in personalized asthma management strategies.

Reproducibility of the phenotype, based on the sputum cell counts in asthma, has been investigated in a small number of studies. Two earlier studies conducted in adult asthmatics report stability of the sputum phenotype over a 5-year follow-up [7, 8]. However, in the study of Simpson et al. [7] 17.5% of 40 subjects changed inflammatory phenotype between EA and NEA over short-term evaluation [7]. In the study of van Veen et al., 30% of subjects, classified as EA at baseline, changed inflammatory phenotype on the second visit after 5 years, although authors admit that assessments were performed during natural course of the disease, without planned interventions and despite variations in exposure to asthma triggers [8]. Several recent studies challenge the view that phenotypic classification of asthma according to induced sputum analysis is stable over time [9–11]. In one of these studies authors examined prospectively sputum profiles of moderate and severe asthmatics (some of them treated with OCS) using multiple samples over 1-year period. Stable phenotypes were noted in only one-third of subjects [9]. Another study examined stability of asthma phenotypes over time and with different treatment regimens (placebo, terbutaline, budesonide, and terbutaline and budesonide combination) proving that cellular profile of the sputum samples changed frequently during the course of the study [10]. Of course some variability in sputum cell profile was expected, for example, ICS treatment decreasing eosinophil count; however authors observed considerable instability of phenotype between the steroid withdrawal and placebo periods where there were no differences in treatment. In a most recent study 40 steroid-naïve asthmatics with NEA were randomized to receive salmeterol or fluticasone and sputum analysis was performed 3 times over 6 months [11]. Results showed that 40% of subjects receiving salmeterol had transient sputum eosinophilia indicating that NEA is not stable in all subjects. Instability of the inflammatory phenotype has also been reported in one study conducted in children with asthma. The majority (61%) of studied children demonstrated a change in inflammatory phenotype on repeated assessments over 1-year follow-up [11]. This variability does not appear to be due to changes in ICS treatment, since observed changes in sputum profiles were not associated with changes in doses of ICS, although variable compliance to treatment cannot be excluded.

Our results together with available data from other studies confirming substantial variability of asthma inflammatory phenotypes draw into question the utility of sputum cell profile analysis in individualized asthma management strategies. Sputum induction procedure and subsequent cell analysis remain time-consuming and costly, therefore unlikely to be repeated frequently in routine clinical setting. Our study results and body of data published recently prove that single sputum sample assessment cannot reliably distinguish between EA and NEA and cannot help to guide clinical decisions in asthma patients. This finding leads to the conclusion that asthma inflammatory phenotypes are not stable over time, which stays in line with hallmark of asthma definition such as variability in airway function and symptoms. It is expected that underlying chronic inflammatory process will also vary.

It is well known that variation in allergen exposure, change in disease control or treatment, and airway infections may be responsible for phenotypic instability. In our study we paid a special attention to exclude confounding factors in assessment of reproducibility of inflammatory phenotypes. Namely, all subjects were asked to fill in ACQ questionnaire before sputum induction procedure at each study visit to make sure that there was no change in disease control between visits. Furthermore antiasthmatic therapy remained stable between study visits and exacerbation during visits interval was an exclusion criterion. Such an approach minimizes the likelihood of the influence of external factors on inflammatory phenotype changes. Our study is the first one which evaluated reproducibility of sputum cell profiles in 3 different groups of an adult asthma cohort: SN not receiving ICS, MMA receiving regular ICS treatment, and RA receiving OCS treatment. This provides insight into stability of phenotypes in wide spectrum of asthmatics. Most previous studies assessing sputum phenotypes enrolled only asthmatics treated with ICS/OCS [7–10, 12]; therefore there is scarce of data on reproducibility of sputum profiles in SN group of asthmatics [11].

The main limitation of our study is relatively small sample size, especially when evaluating each group of asthmatics separately. To determine possible mechanism of phenotype instability in asthma it would be important to include more patients and to perform prospective studies examining more closely variability in sputum cell profiles.

## 5. Conclusion

Inflammatory phenotype classification using induced sputum is not fully reproducible in adults with asthma in short-term evaluation. EA phenotype seems to be more stable across all groups of asthmatics whereas NEA phenotype remained stable only in RA group. The obtained results question possible usefulness of sputum profiles in asthma management.

## Figures and Tables

**Table 1:** Characteristic of study participants: steroid-naïve asthma (SN), mild to moderate asthma (MMA), and refractory asthma (RA) cohorts. Values are presented as mean ± SD.

	SN group	MMA group	RA group
*N*	17	33	16
Age (years)	33.41 ± 11.26	47.52 ± 13.9^***^	50.5 ± 9.96^***^
Sex F : M	11 : 6	16 : 17	11 : 5
History of asthma (years)	5.96 ± 8.86	10.24 ± 12.9	23.68 ± 11.3^***^
Positive SPT (*N*)	14	21	16
FEV_1_ (actual/% of predicted value)	3.32 ± 0.91/92.4 ± 13.9	2.69 ± 0.86^*^/83.06 ± 14.7	1.79 ± 0.61^***^/58.94 ± 11.8
FEV_1_/FVC (%)	73.35 ± 8.19^***^	70.6 ± 8.6	61.63 ± 11.48^**^
Mean dose of ICS (*µ*g)^†^	0	1373.12 ± 908.8	1307.5 ± 586
Mean dose of OCS (mg)^‡^	0	0	17.75 ± 7.96

SPT, skin prick tests; FEV_1_, forced expiratory volume in one second; FVC, forced vital capacity; ICS, inhaled corticosteroids; OCS, oral corticosteroids.

^†^Equivalent to CFC-beclomethasone dipropionate.

^‡^Equivalent to prednisone.

*P* values: age: ^***^ *P* < 0.001 versus SN; history of asthma: ^***^ *P* < 0.001 versus SN and MMA; FEV_1_: ^*^ *P* < 0.05 versus SN, ^***^ *P* < 0.001 versus SN and MMA; FEV_1_/FVC: ^**^ *P* < 0.01 versus MMA, ^***^ *P* < 0.001 versus RA.

**Table 2:** The cellular profile of sputum, asthma phenotypes, and ACQ score at visit 1. Values are presented as mean ± SD.

	SN group	MMA group	RA group
Sputum cytology			
Total cells (×10^6^/mL)	5.41 ± 7.47	4.96 ± 4.13	5.04 ± 4.0
Viability (%)	81.10 ± 10.67	80.6 ± 11.31	80.9 ± 12.6
Squamous cells (%)	5.89 ± 5.74	6.85 ± 10.8	9.31 ± 7.97
Epithelial cells (%)	0.85 ± 0.52	1.1 ± 0.7	0.62 ± 0.49
Macrophages (%)	59.03 ± 18.57	56.2 ± 21.6	59.25 ± 15.4
Lymphocytes (%)	4.99 ± 6.44	4.79 ± 6.34	6.56 ± 3.25
Neutrophils (%)	27.12 ± 18.8	24.15 ± 20.1	21.94 ± 15.31
Eosinophils (%)	3.05 ± 3.89	7.9 ± 14.57	2.75 ± 3.61
Inflammatory phenotype, *n* (%)			
Eosinophilic	8 (47%)	18 (55%)	7 (44%)
Neutrophilic	1 (6%)	0	0
Paucigranulocytic	8 (47%)	14 (42%)	9 (56%)
Mixed granulocytic	0	1 (3%)	0
ACQ score	0.96 ± 0.58	1.31 ± 1.06	2.4 ± 0.81^***^

SN, steroid-naïve asthma; MMA, mild to moderate asthma; RA, refractory asthma. ^***^ *P* < 0.001 versus MMA group and SN group.

**Table 3:** The cellular profile of sputum, asthma phenotypes, and ACQ score at visit 2. Values are presented as mean ± SD.

	SN group	MMA group	RA group
Sputum cytology			
Total cells (×10^6^/mL)	5.41 ± 7.47	7.21 ± 15.9	5.18 ± 4.14
Viability (%)	81.78 ± 13.72	81.9 ± 10.1	80.52 ± 10.76
Squamous cells (%)	4.36 ± 4.21	6.92 ± 7.33	8.31 ± 6.04
Epithelial cells (%)	0.9 ± 0.67	0.7 ± 0.52	1.1 ± 0.78
Macrophages (%)	60.42 ± 14.47	53.45 ± 20.52	57.25 ± 10.91
Lymphocytes (%)	4.03 ± 3.4	5.83 ± 8.63	6.75 ± 4.21
Neutrophils (%)	26.48 ± 16.78	28.35 ± 21.52	25.25 ± 14.65
Eosinophils (%)	4.68 ± 6.42	5.03 ± 6.52	2.5 ± 2.94
Inflammatory phenotype, *n* (%)			
Eosinophilic	10 (59%)	19 (58%)	7 (44%)
Neutrophilic	1 (6%)	1 (3%)	1 (6%)
Paucigranulocytic	6 (35%)	11 (33%)	8 (50%)
Mixed granulocytic	0	2 (6%)	0
ACQ score	0.9 ± 0.59	1.37 ± 1.05	2.48 ± 0.89^***^

SN, steroid- naïve asthma; MMA, mild to moderate asthma; RA, refractory asthma. ^***^ *P* < 0.001 versus MMA group and SN group.
